# Physiological and Behavioral Responses to Vocalization Playback in Mice

**DOI:** 10.3389/fnbeh.2020.00155

**Published:** 2020-09-01

**Authors:** Alexandra C. Niemczura, Jasmine M. Grimsley, Chae Kim, Ahmad Alkhawaga, Austin Poth, Alyssa Carvalho, Jeffrey J. Wenstrup

**Affiliations:** ^1^Department of Anatomy and Neurobiology, Northeast Ohio Medical University, Rootstown, OH, United States; ^2^School of Biomedical Sciences, Kent State University, Kent, OH, United States; ^3^Brain Health Research Institute, Kent State University, Kent, OH, United States

**Keywords:** anxiety, communication, corticosterone, mouse, stress, ultrasonic, vocalization, low frequency

## Abstract

In mice, the caller’s production of social vocalizations has been extensively studied but the effect of these vocalizations on the listener is less understood, with playback studies to date utilizing one vocalization category or listeners of one sex. This study examines how several categories of mouse vocalizations affect listeners of both sexes to better understand the communicative functions of these vocal categories. We examined physiological and behavioral responses of male and female CBA/CaJ mice to playback of four social vocalization categories: ultrasonic vocalizations (USVs), low-frequency harmonic calls, mid-frequency vocalizations, and noisy calls. Based on the conditions under which these calls are emitted, we hypothesized that playback of these vocal categories would have differential effects on the listeners. In females, playback of all four vocalization categories increased stress hormone levels (corticosterone), but only the non-USV categories increased corticosterone in males. The magnitude of corticosterone increase in non-USV trials was greater in females than in males. In open field tests, all four vocal categories decreased central ambulation in males and females, indicating an increase in anxiety-related behavior. Further, we found that the proportions of USVs emitted by subjects, but not their overall calling rates, were affected by playback of some vocal categories, suggesting that vocalization categories have different communication content. These results show that, even in the absence of behavioral and acoustic contextual features, each vocal category evokes physiological and behavioral responses in mice, with some differences in responses as a function of the listener’s sex and playback signal. These findings suggest that at least some of the vocal categories have distinct communicative functions.

## Introduction

The social vocalizations of mice, like those of humans and other vertebrates, reflect the internal state of the sender and influence the internal state and behavior of the listener. There has been an extensive study in mice of the caller’s production of vocalizations, focusing on the behavioral contexts within which the vocalizations are emitted (Nyby, 1983; Maggio and Whitney, 1985; Holy and Guo, 2005; Wang et al., 2008; Williams et al., 2008; Grimsley et al., 2011, 2016; Hanson and Hurley, 2012; Sangiamo et al., 2020) and how vocal behavior is altered by internal state (Gaub et al., 2016; Grimsley et al., 2016; Demir et al., 2020) and in disease models (Scattoni et al., 2008; Wöhr et al., 2011; Belagodu et al., 2016). However, few studies have examined the effect of mouse vocalizations on the listener. Playback studies to date are limited in number and scope, mostly utilizing one vocalization category (Chen et al., 2009; Hammerschmidt et al., 2009) or only male (Grimsley et al., 2013) or female listeners (Pomerantz et al., 1983; Hammerschmidt et al., 2009; Asaba et al., 2017). This study examines physiological and behavioral responses to four categories of mouse vocalizations in both males and females, to better understand the communicative functions of these vocal categories.

**Figure 1:**
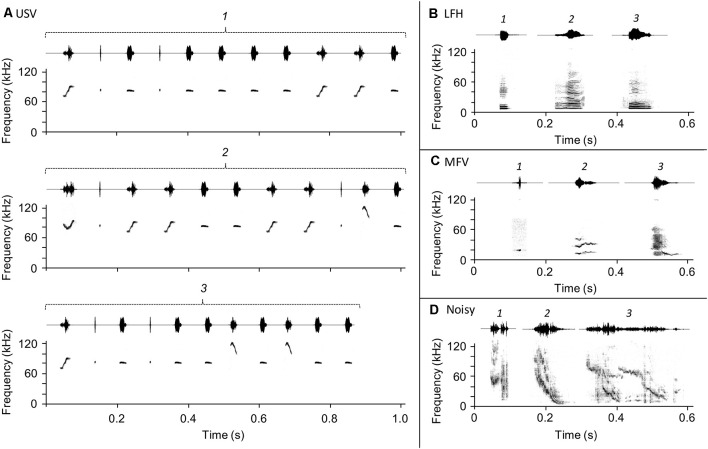
Vocalization stimuli used in the playback experiment. Three exemplars (labeled *1*, *2*, and *3*) of each vocalization category were presented in pseudorandom sequences throughout the playback period. **(A)** Ultrasonic vocalizations (USVs), **(B)** low-frequency harmonic call (LFH), **(C)** mid-frequency vocalization (MFV), **(D)** Noisy call.

*Ultrasonic vocalizations* (USVs), the most common category emitted by adult mice, have a fundamental frequency greater than 20 kHz (Figure 1A) and a variety of spectrotemporal patterns that range from simple to complex (Sales née Sewell, 1972; Holy and Guo, 2005; Portfors, 2007; Hammerschmidt et al., 2009; Musolf et al., 2010; Grimsley et al., 2011, 2016; Arriaga, 2014; Gaub et al., 2016). USVs are emitted in a broad range of social contexts: by pups when isolated from their mothers (Liu et al., 2003; Portfors, 2007; Grimsley et al., 2011), by males and females during courtship and mating interactions (Wang et al., 2008; Lahvis et al., 2011; Neunuebel et al., 2015), and by males and females during voluntary interactions with same-sex conspecifics (Moles et al., 2007; Wang et al., 2008; Grimsley et al., 2011). Evidence suggests that USVs indicate a positive affective state in male callers (Wang et al., 2008) but maybe stressful or associated with the negative state for females during mating situations (Pomerantz et al., 1983; Hammerschmidt et al., 2009; Chabout et al., 2015). USVs are thus highly relevant communication calls for females that may elicit behavioral and stress hormonal change. In males, the behavioral or endocrine responses evoked by USVs in listening animals is less clear.

*Low-frequency harmonic calls* (LFHs) are harmonic stacks of a mostly flat frequency-time profile with fundamental frequencies below 5 kHz (Figure 1B; Grimsley et al., 2011), audible to humans as the mouse “squeak” (Williams et al., 2008). LFH calls are emitted by females during mating when attacked or mounted by males (Sales née Sewell, 1972, “audible cries”; Grimsley et al., 2013; Keesom and Hurley, 2016) and by both sexes in response to acute pain (Williams et al., 2008), during fighting (Gourbal et al., 2004), and during restraint stress (Grimsley et al., 2016). Although most evidence suggests that LFHs indicate a negative affective state in the caller, the effect for a listener can be modulated by olfactory cues (Grimsley et al., 2013). We hypothesized that outside of a mating context, LFH calls elicit stress in male and female listeners.

*Mid-frequency vocalizations (MFVs)* were recently identified by Grimsley et al. (2016). They are tonal vocalizations with a fundamental frequency between 9 and 15 kHz (Figure 1C). MFVs are typically emitted by mice undergoing several types of restraint, where they comprise about 15% of vocalizations and are associated with elevated stress levels (Grimsley et al., 2016). Mouse responses to playback of the MFV have not been assessed; we hypothesize that playback of the MFV evokes stress and is aversive to mice.

*Noisy calls*. Noisy vocalizations are structured, usually, frequency-modulated calls that span a wide frequency range (10–120 kHz) and have chaotic elements (Figure 1D). They are exclusively emitted by adults (Grimsley et al., 2011), frequently during isolation (43% of vocalizations) but less commonly during restraint (8%) or mating (1%; Grimsley et al., 2016). Grimsley and colleagues suggest that, since they are emitted in isolation and span a wide range of frequencies, noisy vocalizations may be “seeking” signals. To our knowledge, noisy vocalizations have not been used in playback studies. Since noisy vocalizations may be an affiliative, “seeking” call, we hypothesized that playback evokes stress in listening mice.

To test how these vocal categories affect internal state and behaviors in mice, we presented simple elements of these categories in males and females while monitoring corticosterone levels, locomotor behavior, and vocal behavior.

## Materials and Methods

### Animals

Subjects were sexually naïve, adult male (*n* = 12) and female (*n* = 12) CBA/CaJ mice (JAX^®^; Bar Harbor, ME, USA), with ages between P90 and P270. This strain is commonly used in auditory research because these mice maintain normal hearing thresholds throughout most of their lifespan (Ohlemiller et al., 2010). Animals were pair-housed under a 12 h reverse light/dark cycle (ZT0, 10 pm) and received food and water *ad libitum*. The female estrous cycle was assessed visually on experiment days, as described by Byers et al. (2012). All experiments took place during the dark phase, between ZT15 and ZT17 (1:00–3:00 pm). Mice were habituated to the testing room for 2 h before the first experiment. All procedures were approved by the Institutional Animal Care and Use Committee at Northeast Ohio Medical University (NEOMED).

### Vocalization Stimuli

Vocalization stimuli were three typical exemplars from each of four categories of social vocalizations (USV, MFV, LFH, Noisy; Figure 1) obtained from mice in previous studies (Grimsley et al., 2011, 2016). These recorded mice were unknown and not closely related to the subjects of the current study. All vocal exemplars were selected on the bases of their high signal-to-noise ratios and statistical features, lying within one standard deviation of mean values for duration, harmonics, frequency, and frequency modulation of all recorded syllables from adults for the category. The USVs were recorded from three adult mice (P90–P120) during male-female interactions before mounting (Grimsley et al., 2011); they were likely emitted by males (Wang et al., 2008). Bouts of USVs (Figure 1A) were selected by the “virtual mouse vocal organ,” which created sequences from pre-recorded tonal USVs in a pattern and with inter-syllabus intervals that are typical for adult males (Grimsley et al., 2011). LFHs were likely recorded from females during mating, or during agitation (Grimsley et al., 2011). MFVs were recorded from 14 animals in a jacket restraint context (Grimsley et al., 2016), while Noisy vocalizations were recorded from animals in isolation (Grimsley et al., 2016). The sex of the recorded mice who produced test MFV and Noisy calls is not known.

Vocalization stimuli were played using a speaker (LCY-K100, Ying Tai Audio Company, Hong Kong) located outside of a hole in the side of the arena. Signals were compensated to account for the high-frequency roll-off the speaker in use. All stimuli were presented at a target level of ~80 dB SPL (15 cm from the speaker). For USVs, this is approximately 10 dB greater than the level at which they are emitted during mating (Lahvis et al., 2011). For non-USVs, this is about 10 dB less than the level at which they are typically emitted in our laboratory (unpublished data). The uniform peak sound level was chosen to control for physiological or behavioral responses that could result solely from sound level differences (Gadziola et al., 2016). Each recorded exemplar contained a low level of background noise. We place a 10-ms ramp at the beginning and end of the sound files to ensure that no artifact was created by sudden onset of the background noise. We observed no startle responses to these stimuli, although animals would often orient toward the speaker after sound onset. Animals were placed in the arena before the onset of the sound stimulus. During experimental trials, the three exemplars of a single vocalization category were played in a pseudorandom order throughout the last 20 min of the session (one exemplar every 4 s). Vocalizations were started 10 min after mice were placed in the arena to avoid having the response to a novel environment as a confounding factor. We did not include a non-vocalization acoustic signal in our stimulus set because the affective nature of such stimuli is uncertain and because our focus was on a comparison of responses to the different vocal categories.

### Experimental Design

**Figure 2:**
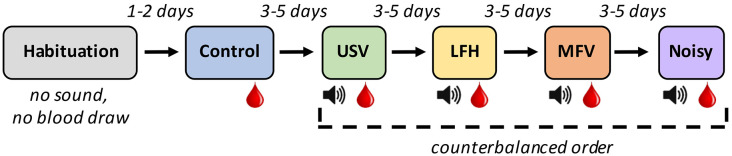
Experimental design for playback experiment: Six 30-min trials of different types were experienced by each animal. The* Red drop* represents trials after which blood was drawn. The *Speaker symbol* represents trials in which the animal was exposed to exemplars of the indicated vocalization category.

To assess physiological (hormonal), locomotive, and vocal responses of mice to playback of four vocalization categories, animals individually underwent six 30-min sessions (Figure 2) in an open-field arena. The first session was the habituation trial, in which an animal acclimated to the arena in the absence of vocalization stimuli. Two days later, the animal underwent the control trial, in which no vocal stimuli were played. The animal’s baseline locomotive and vocal behaviors and corticosterone levels were assessed at that time. Three to five days later, the animal underwent the first experimental trial, in which one of the four categories of vocalizations was presented. During the session, the animal underwent 10 min of acclimation, followed by 20 min of playback. Three to five days after that, the animal underwent the second experimental trial, in which a different vocalization category was presented. This process was repeated for the remaining two vocalization categories. The order in which the experimental trials occurred was counterbalanced across subjects.

### Arena

**Figure 3:**
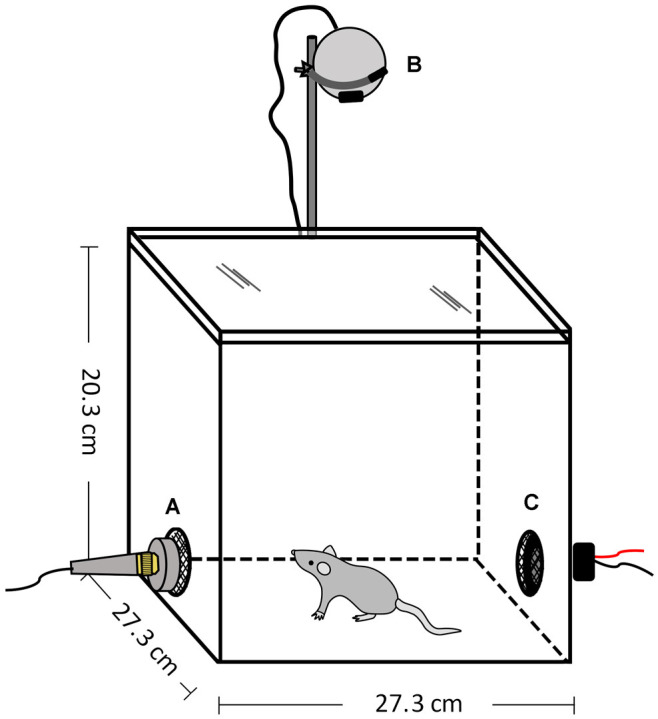
Open field arena to assess responses to vocalization playback. **(A)** Microphone, **(B)** Video camera, **(C)** Speaker.

All experiments occurred within an opaque, white Plexiglas^®^ chamber (27.3 cm W × 27.3 cm D × 20.3 cm H) with a transparent, colorless lid (ePlastics^®^, San Diego, CA, USA; Figure 3). The arena was housed within a sound-attenuating chamber (Industrial Acoustics Company, Bronx, NY, USA) and illuminated by dim red light (~30 lux). There were two holes (5.08 cm diameter) located in walls on opposite sides of the arena at the approximate height of the mouse’s head: one for the speaker to play the vocalization stimuli and the other for a microphone to record the animal’s vocal behavior. The holes were covered with a black metal mesh, so that both sides of the arena were visually similar. Speaker and microphone placement were interchanged between trials and the animal was placed in the center of the arena at the start of each session. The arena was cleaned with 70% EtOH between sessions.

### Measurements and Analysis

#### Assessment of Anxiety-Related Behavior

Central ambulation, an inverse measure of anxiety-related behavior, was assessed during the first 10 min of playback of each vocalization category. Animal location and movement were recorded with an overhead-mounted camera and then analyzed using a custom-written DataWave experiment in a semi-automated process (DataWave Videobench 7.0; DataWave SciWorks, Loveland, CO, USA). The arena was first divided into two equally sized zones, a square center and surrounding perimeter (every 373 cm^2^), which were superimposed by the investigator onto each recorded video. A cardboard template was taped to the computer screen and used as a stencil for accurate superimposition of the zone boundaries. The investigator then selected a distinctly colored spot on the center of the animal’s head, to be tracked by the program using color detection. An automated process was used to track the amount of movement of the selected spot within each of the zones during the first 10 min of vocalization playback. The automated process converted the distance traveled by the selected spot from pixels to centimeters. The automated analysis was monitored by the investigator throughout each trial to ensure accurate detection of the spot on the animal’s head. All video files were analyzed by a single investigator and all analyses for this study took place under single-blind conditions. The proportion of central ambulation was calculated by dividing the distance traveled in the center zone by the total distance traveled (the sum of distance traveled in both zones) for a given trial. For each individual, central ambulation during the first 10 min of vocalization playback in experimental trials was compared to central ambulation during the corresponding period in the no-sound control trial. This measure was used because it controls for individual differences in total ambulation. A lower proportion of central ambulation indicates an increase in anxiety-related behavior (Kulesskaya and Voikar, 2014).

#### Analysis of Vocal Behavior

Vocal behavior was recorded using an ultrasonic condenser microphone (CM16/CMPA, Avisoft Bioacoustics, Berlin, Germany) and was analyzed using Avisoft Bioacoustics SAS lab software. Markers indicating the start and end of each emitted vocalization and a label indicating the vocalization type (based on category descriptions by Grimsley et al., 2016) were inserted manually. Data were then extracted automatically from Avisoft for syllable duration, peak frequency, and the number of instances of vocalization type. For the first 10 min of playback in each trial, we assessed both the number of vocalizations and the proportion of USVs emitted by experimental subjects while listening to the playback. USV proportion, calculated by dividing the number of USVs emitted by the total number of vocalizations emitted, provided an additional measure of vocal behavior beyond USV calling rate (e.g., Scattoni et al., 2008) or the total number of vocalizations emitted (e.g., Grimsley et al., 2016), including each of the non-USV social vocalizations emitted by CBA/CaJ mice.

#### Plasma Corticosterone

Thirty minutes after vocalization stimulus onset, and at the corresponding time point for the no-sound control trial, animals were lightly anesthetized with 4% Isoflurane (Abbott Laboratories, Abbott Park, IL, USA) and blood samples were collected by a submandibular puncture. Blood samples were allowed to clot at room temperature for 1 h and then were centrifuged (3,500 rpm) at 4°C for 1 h. The separated plasma was stored at −80°C until ready for assay (Jasnow et al., 2000). The samples were diluted (1:100) and placed in a 70°C water bath for 1 h to separate corticosterone from corticosterone binding globulin (J. Johnson, personal communication, June 6, 2018). Plasma corticosterone levels were determined using Corticosterone Enzyme-Linked Immunosorbent Assay (ELISA; Enzo^®^, Farmingdale, NY, USA) and were assessed at 450 nm without correction. Higher corticosterone levels indicate greater physiological stress (Ahn et al., 2016).

### Statistical Analyses

Statistical analyses examined the effects and interactions associated with the independent variables of trial type and sex. The dependent variables were plasma corticosterone, central ambulation, the total number of vocalizations emitted, and USV proportion. *P*-values for the four main analyses (two-way ANOVAs) were Bonferroni-corrected and were statistically significant at *α* < 0.0125. All statistical analyses were conducted in SPSS. Outliers were assessed with boxplot analysis. Data points that were greater than 1.5 box-lengths from the edge of the box were removed from that specific analysis. Due to the repeated measures design, all other data for the “outlier” animals were removed from the same analysis. The data from at most one animal were removed from each analysis; these are indicated in the appropriate figure legends. To assess whether the female estrous stage was associated with corticosterone, central ambulation, or vocal behavior, we performed point biserial correlations in SPSS. In all figures, error bars represent the standard error of the mean (SEM).

## Results

These Results describe several measures of response to social vocalizations in male and female mice. For each of the four categories of social vocalizations, we describe how the vocal stimulus affected plasma corticosterone levels, central ambulation, and vocalizations by the listening mouse. We show that: (1) there are sex differences in the plasma corticosterone levels evoked by vocalization playback; (2) that all call types evoke anxiety-like behavior in males and females; and (3) the vocal behavior of both sexes differed depending on the vocalizations being presented.

### Plasma Corticosterone Levels

**Figure 4:**
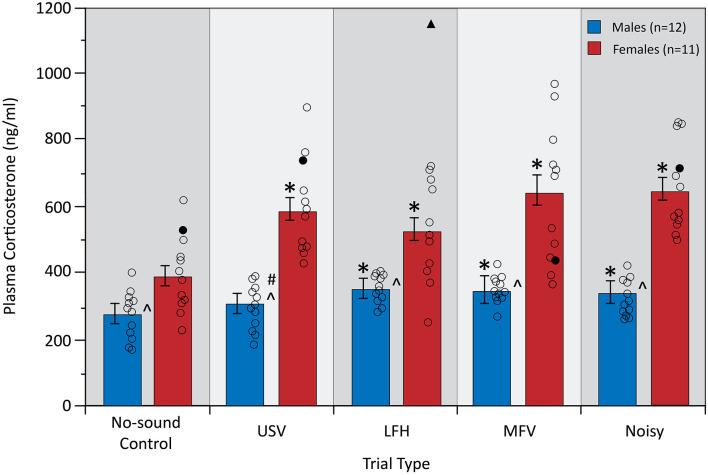
Plasma corticosterone levels in response to vocalization playback. All vocalization categories increased corticosterone in females, but only non-USV categories increased corticosterone in males. Females had a greater plasma corticosterone concentration than males across trials, and the magnitude of increase was greater in females than in males. Error bars represent standard error of the mean (SEM). Symbols for Figures 4–7: *differs from no-sound control, *p* < 0.05; ^∧^differs between sexes, *p* < 0.0125; ^#^interaction between sex and trial type, *p* < 0.0125; unfilled black circles represent individual data points for males and females. Black triangle indicates an outlier data point from one animal that was removed from this analysis; filled black circles represent non-outlier data from the same animal that was also removed due to the repeated measures tests.

Plasma corticosterone, a physiological measure of stress, was assessed after five trials: a no-sound control trial and four vocalization playback trials (Figure 4). A two-way mixed ANOVA yielded a significant interaction between trial type and sex on corticosterone (*F*_(4,84)_ = 5.19, *p* = 0.001, partial *η*^2^ = 0.198), indicating that the physiological stress response to the vocalization categories differed between males and females. Simple main effects analysis revealed that USVs evoked an increase in corticosterone from no-sound control in females (*p* = 0.001), but not in males (*p* = 0.230). For females, corticosterone increased from no-sound control for all four vocalization categories (USV, *p* = 0.001; LFH, *p* = 0.031; MFV, *p* = 0.001; Noisy, *p* < 0.001). For males, corticosterone increased from no-sound control for LFH (*p* = 0.013), MFV (*p* = 0.004), and Noisy (*p* = 0.046) categories. These results indicate that USVs evoked physiological stress in females but not in males.

There was a main effect of sex (*F*_(1,21)_ = 47.68, *p* < 0.001, partial *η*^2^ = 0.694), with females showing greater corticosterone than males across all trials (*p* < 0.001). Interestingly, females also showed a greater magnitude of corticosterone increase from control for all four vocalization categories, relative to males, (*p* < 0.05). Corticosterone was not affected by trial order (*p* > 0.05) or by estrous stage in females (*p* > 0.05). Corticosterone did not correlate with the total distance traveled (*p* > 0.05), indicating that the stress response cannot be explained by differences in total ambulatory activity.

### Locomotory Behavior

**Figure 5:**
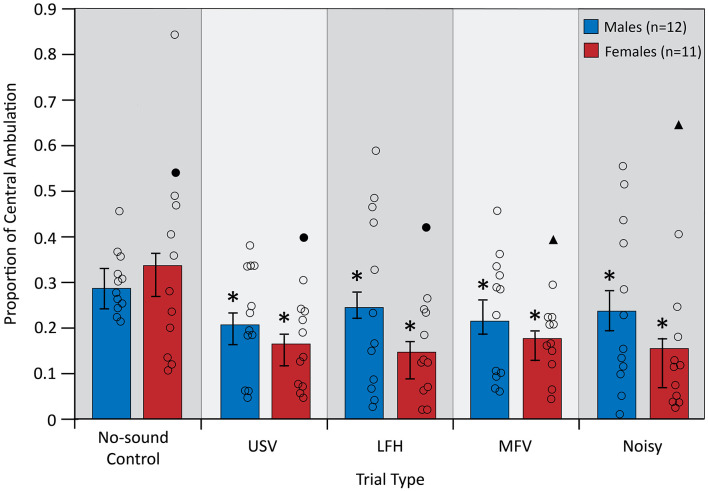
Central ambulation decreased from no-sound control for all vocalization categories. For symbols, see Figure 4. Outlier data excluded from this analysis (filled triangles and circles) were not from the same animal as in Figure 4. *Differs from no-sound control, *p* < 0.05.

Central ambulation, an inverse measure of anxiety-related behavior, was assessed during five trials: a no-sound control trial and four vocalization playback trials. An “anxious” mouse would be expected to demonstrate lower central ambulation (Carola et al., 2002; Seibenhener and Wooten, 2015). A significant main effect of trial type on central ambulation indicated that anxiety-related behavior differed between trial types (*F*_(4,84)_ = 4.135, *p* = 0.004, partial *η*^2^ = 0.165; Figure 5). Planned contrasts revealed that playback of all vocalization categories decreased central ambulation from control (USV > control, *p* = 0.009; LFH > control, *p* = 0.012; MFV > control, *p* = 0.012; Noisy > control, *p* = 0.011), indicating that all vocalization categories increase anxiety-related behavior. There was not a main effect of sex (after correcting *p-values* for multiple comparisons), indicating that anxiety-related behavior did not differ between males and females (*F*_(1,21)_ = 4.772, *p* = 0.040, partial *η*^2^ = 0.185). There was not a significant interaction of trial type and sex (*F*_(4,84)_ = 1.369, *p* = 0.252, partial *η*^2^ = 0.061), indicating that anxiety-related behavior is not differentially affected in males and females. Anxiety-related behavior was not affected by trial order (*p* > 0.05) or by estrous stage in females (*p* > 0.05).

### Vocal Behavior

**Figure 6:**
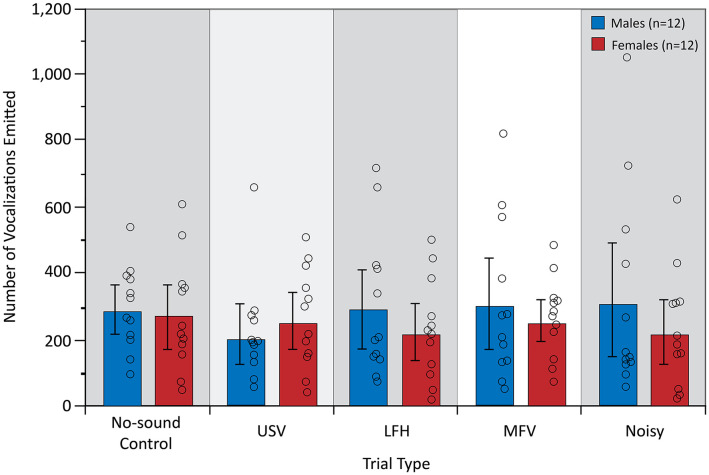
The total number of vocalizations emitted during the first 10 min after stimulus onset. There were no significant differences in vocalizations for trial type. For symbols, see Figure 4.

**Figure 7:**
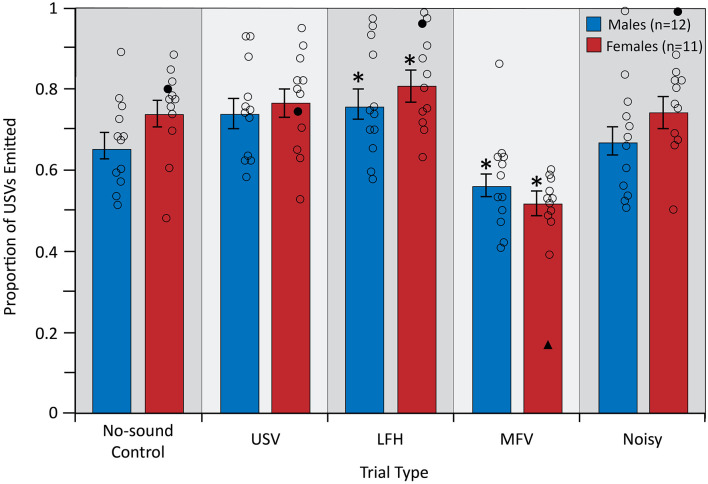
The proportion of emitted USVs changed in LFH and MFV playback trials. For symbols, see Figure 4. Outlier data excluded from this analysis (filled triangles and circles) were not from the same animal as in Figures 4 or 5. *Differs from no-sound control, *p* < 0.05.

The analysis of vocal behavior was based on 31,984 total vocalizations (17,137 for males and 14,847 for females). The total number of vocalizations emitted, and therefore the vocalization rate over 10 min, did not differ among trial types (*F*_(4,84)_ = 0.609, *p* = 0.657, partial *η*^2^ = 0.009) or between sexes (*F*_(1,21)_ = 0.780, *p* = 0.387, partial *η*^2^ = 0.024; Figure 6). However, there was a significant main effect of trial type on the proportion of USVs emitted by the listener (*F*_(4,84)_ = 25.953, *p* < 0.001, partial *η*^2^ = 0.553), indicating that vocal behavior differed across trial types (Figure 7).

Planned contrasts revealed that during playback of LFHs, both male and female subjects emitted an increased proportion of USVs (*p* = 0.010). In contrast, during playback of MFVs, both male and female subjects emitted a reduced proportion of USVs (*p* < 0.001). This reduced USV emission was countered by increased production of MFV calls. That is, both males and females emitted a greater proportion of MFVs during the MFV playback trial than in other trials. For males, 41.6% of all vocalizations emitted in the MFV playback trials were MFVs, compared to 18.4–30.4% in the other trials. For females, 41.3% of all vocalizations emitted in the MFV playback trials were MFVs, compared to 13.3–18.3% in the other trials.

Neither USVs nor Noisy playback had a significant effect on USV proportion (*p* = 0.023 and *p* = 0.750, respectively), after correcting the *p*-value for multiple comparisons (Figure 7). There was no main effect of sex on emitted USV proportion (*F*_(1,21)_ = 0.906, *p* = 0.352, partial *η*^2^ = 0.041), indicating that USV proportion did not differ between males and females. Further, there was no significant interaction between sex and trial type (*F*_(4,84)_ = 1.797, *p* = 0.137, partial *η*^2^ = 0.079), indicating that USV proportion is not differentially affected by playback of the four vocalization categories in males and females. USV emission was not affected by trial order (*p* > 0.05) or by estrous stage in females (*p* > 0.05).

Overall, our sample size of non-USVs was not sufficiently large to perform *post hoc* analyses comparing the emission of each of the four vocalization categories in the four vocalization playback contexts.

## Discussion

Mice emit at least four categories of social vocalizations under a variety of behavioral contexts—USVs and three categories of lower frequency and mostly broadband vocalizations including LFH calls, MFVs, and Noisy calls. This study asked a basic question: do the different categories of social vocalizations themselves differentially affect listeners, apart from the social context and more complex acoustic sequences that occur naturally? If so, this implies that the vocal categories themselves have meaning. To assess these effects on the listeners, we used three assays: corticosterone levels as a measure of physiological stress, central ambulation as a measure of anxiety, and vocal responses as a measure of the social communication content of received vocal signals. There were several general findings. First, playback of all four vocalization categories increased corticosterone levels in females, but only non-USV categories increased corticosterone in males. The magnitude of corticosterone increase in non-USV trials was greater in females than in males. Second, all four vocalization categories decreased central ambulation, indicating an increase in anxiety-related behavior. Finally, we found that the proportions of USVs emitted by subjects, but not their overall calling rates, were affected by playback of some vocal categories. These results show that, even in the absence of behavioral and acoustic contextual features, each vocal category evokes physiological and behavioral responses in mice. The results further suggest that at least some vocal categories may have distinct communicative functions and that the communicative function of some signals may be influenced by the listener’s sex.

### USV Playback

USVs are emitted in a variety of social interactions by both sexes, but predominantly by males during mating (Moles et al., 2007; Wang et al., 2008; Grimsley et al., 2011; Lahvis et al., 2011; Arriaga, 2014; Neunuebel et al., 2015; Heckman et al., 2017; Sangiamo et al., 2020). We, therefore, hypothesized that USV playback would evoke differential responses by males and females. It is noteworthy that our USV stimuli are representative of those emitted by males during low-intensity male-female interactions, distinct in many features from USVs produced near the time of copulation (Hanson and Hurley, 2012; Gaub et al., 2016; Ghasemahmad et al., 2019). Nonetheless, we found that playback of these bouts of USVs evoked stress responses in females but not males. Other studies have also observed sex-based effects of USV playback. For example, Hammerschmidt et al. (2009) found that USVs evoke approach behavior in females only, while Tsukano et al. (2015) reported greater auditory cortical response amplitude to USVs in females compared to males. Our results support previous suggestions that USVs are emitted by males as a “courtship” signal (Wang et al., 2008; Lahvis et al., 2011) and therefore, have differing communicative value females and males. However, the increased anxiety-related measure in males supports the conclusion that USVs have salience for both sexes.

Overall, the proportion of USVs emitted was not influenced by USV playback in males or females, suggesting that in the absence of non-auditory cues or more extended vocal sequencing, USVs do not call for a particular vocal response from the listener. With the addition of non-auditory cues and mating behavior, however, both male and female vocalizations change (Hanson and Hurley, 2012; Grimsley et al., 2013).

### LFH Playback

In the relatively neutral behavioral context used here, we found that LFH calls increased corticosterone levels and decreased central ambulation in both sexes. This result is consistent with a previous study by Chen et al. (2009) showing that playback of LFH calls increased the heart rate of listening mice and may serve as a “distress” signal. This category, when presented in the absence of salient non-auditory cues, may function as an “alarm call,” which warns the listener of impending danger, such as a predator or a competitor, and evokes a stress response to initiate the appropriate behavioral response (Smith, 1965). However, both the salience and valence of the LFH call depend on context. While the stress and behavioral responses reported here and cited above suggests a negative interpretation of the call by both male and female listeners, it is substantially more aversive to male listeners when paired with the visual and olfactory signals of cat fur (Grimsley et al., 2013). Further, this pairing is also associated with changes in basolateral amygdalar auditory responses like what occurs during auditory fear conditioning (Grimsley et al., 2013). Beyond salience, the valence of male listeners’ interpretations of the LFH calls changes to attractive when paired with female urine (Grimsley et al., 2013).

LFH playback increased USV emission in both male and female listeners. USVs are emitted in a wide range of positive and negative social interactions (see above) and this result suggests that USVs are the “appropriate” communicative response to LFHs, even in females. This is a puzzling result that should be investigated further, for example, by examining whether this response is modulated by non-auditory sensory cues.

### MFV Playback

MFVs are emitted primarily during restraint, a situation that increases both stress and anxiety in mice (Grimsley et al., 2016). The response of mice to MFV playback similarly evokes increased corticosterone levels and anxiety-related behavior in both males and females. We conclude that this call has a clear negative valence that is recognized by animals that have not experienced the type of extended restraint that resulted in MFV emission. Our findings are consistent with the suggestion by Grimsley et al. (2016) that MFVs have functional similarities to the rat 22 kHz call, which evokes stress in the listener (Sadananda et al., 2008). MFVs may serve as an “alarm” call to activate the listener’s hypothalamic-pituitary-adrenal system in preparation for impending danger. In this case, where the MFV has a clearly negative valence, the observed increase in non-USV vocalizations by the listener (mostly MFVs) seems to be the appropriate communicative response to hearing MFV signals.

Although both LFH and MFV calls are associated with a negative affective state, the differential effect of LFH and MFV playback on emitted USV proportion strongly suggests differential communication function. MFV calls appear to be uniformly associated with a negative behavioral context in vocalizing mice (Grimsley et al., 2016), elicit increased stress and anxiety in both sexes (this study), and evoke increased acetylcholine release in the basolateral amygdala that indicates increased vigilance (Ghasemahmad et al., 2020). LFH calls, by contrast, are produced in a variety of contexts and interpreted by listeners based on other contextual cues, such as odor or their occurrence in mating interactions.

### Noisy Call Playback

Noisy vocalizations are mostly emitted by adult mice during isolation, a situation that involves less physiological stress and anxiety than that which occurs during restraint (Grimsley et al., 2016). It is thus interesting that playback of Noisy calls similarly increased measures of stress and anxiety in listening mice as did the MFV calls. We are unable to explain this response in terms of the “seeking” function proposed by Grimsley et al. (2011, 2016). Nonetheless, the vocal response to Noisy call playback differs from either LFH or MFV playback, suggesting some differences in communicative function. Further work is required to understand the function of Noisy calls.

### Experimental Measures

#### Stress and Anxiety

The greater magnitude of corticosterone response to all vocalizations in females, relative to males, supports previous findings that females show greater activation of the hypothalamic-pituitary-adrenal axis in response to stressors (Kitay, 1961; Handa et al., 1994; Oyola and Handa, 2017). We observed greater corticosterone in females for the control trial as well. This may reflect previous findings that females have greater baseline corticosterone levels than males (Kitay, 1961; Critchlow et al., 1963; Oyola and Handa, 2017), but is unlikely to reflect a sex difference in the response to the open field since animals were previously habituated to the arena. Sex differences in corticosterone were unlikely to have resulted from an interaction between sex and anesthesia and/or blood draw, since the corticosterone response peaks approximately 30 min. after the onset of a stressor. Males showed a similar corticosterone level in USV and control trials, suggesting that repeated exposure to the open-field arena, anesthesia, and/or blood draw did not affect stress level.

Playback of all vocal categories resulted in changes in central ambulation, suggesting increased anxiety-related behavior. This increase generally corresponded to the increased stress measure (corticosterone), but there were a few differences. Thus, there was no sex difference in central ambulation for USVs, and there was no sex difference in the overall size of effects resulting from vocal playback.

#### Vocal Behavior

Previous research demonstrates that vocal behavior is a measure of internal state (Knutson et al., 2002; Sisneros et al., 2004; Burgdorf et al., 2008; Gadziola et al., 2012; Maney, 2013; Wöhr and Schwarting, 2013; Grimsley et al., 2016). We observed no correlation between USV emission and physiological stress level (*p* > 0.05) or anxiety-related behavior (*p* > 0.05), suggesting that there are different mechanisms underlying vocalization production and locomotor and hormonal measures of anxiety and stress. Future studies should investigate additional aspects of vocal behavior, like syllable duration or the presence of harmonics, as they relate to hormonal and behavioral measures of stress and anxiety. It is important to note that the female response to the vocalization categories was not affected by the estrous stage, consistent with previous findings from a USV playback study (Hammerschmidt et al., 2009).

We examined how both the calling rate and call type might be useful in evaluating responses to each vocal signal. Although calling rate, mainly USV rate, has been used to assess the impact of arousal, development, and genetic or alterations (Scattoni et al., 2009: Rotschafer et al., 2012; Gaub et al., 2016), we found that vocal playback of any syllable category did not influence overall calling rate; the rates were the same as in no-sound controls. Instead, we observed that the proportion of emitted non-USV syllables could vary in different directions for different trial types. This indicates that assessment of the entire vocal behavior, not just USV behavior, more effectively captures the emotional affect or communication meaning of the played back calls. Larger datasets are required to assess proportions within the non-USV categories.

In humans as well as experimental animals, the response to vocalizations and other auditory stimuli often goes beyond the analysis of acoustic features. The upper levels of the auditory system and non-auditory areas such as the amygdala assess the significance or meaning, of the sound, which in turn provides the basis for an emotional response to it (Wenstrup et al., 2020). Future research should relate amygdalar and auditory cortical activity to the observed hormonal and behavioral responses to the four vocalization categories.

## Conclusion

In this study, we found differential effects of USV, LFH, MFV, and Noisy playback on the listener, and that the response to USVs depends on sex. This work, combined with previous assessments of the context-based production of vocal categories, suggests differences in the communicative functions of mouse vocalization categories. However, more work is needed to understand these functions, especially for MFV and Noisy categories. Further, these investigations of responses of healthy mice to social vocalizations provide an essential foundation for future studies that can assess how mouse models of autism and related disorders misinterpret vocal meaning.

## Data Availability Statement

The raw data supporting the conclusions of this article will be made available by the authors, without undue reservation.

## Ethics Statement

The animal study was reviewed and approved by the Institutional Animal Care and Use Committee, Northeast Ohio Medical University.

## Author Contributions

AN and JG designed the experiments. AN collected the data. AN, JG, and JW designed analysis. AN, CK, AA, AP, and AC analyzed the data. AN and JW wrote the manuscript. JG edited the manuscript.

## Conflict of Interest

The authors declare that the research was conducted in the absence of any commercial or financial relationships that could be construed as a potential conflict of interest.

## References

[B1] Ahn T. Bae C.-S. Yun C.-H. (2016). Acute stress-induced changes in hormone and lipid levels in mouse plasma. Vet. Med. 61, 57–64. 10.17221/8718-vetmed

[B2] Arriaga G. (2014). “Why the caged mouse sings: studies of the mouse ultrasonic song system and vocal behavior,” in Biocommunication of Animals, ed. Witzany G. (Netherlands: Springer), 81–101.

[B3] Asaba A. Osakada T. Touhara K. Kato M. Mogi K. Kikusui T. (2017). Male mice ultrasonic vocalizations enhance female sexual approach and hypothalamic kisspeptin neuron activity. Horm. Behav. 94, 53–60. 10.1016/j.yhbeh.2017.06.006 28645693

[B4] Belagodu A. P. Johnson A. M. Galvez R. (2016). Characterization of ultrasonic vocalizations of fragile x mice. Behav. Brain Res. 310, 76–83. 10.1016/j.bbr.2016.04.016 27142239

[B5] Burgdorf J. Kroes R. A. Moskal J. R. Pfaus J. G. Brudzynski S. M. Panksepp J. (2008). Ultrasonic vocalizations of rats (*Rattus norvegicus*) during mating, play and aggression: behavioral concomitants, relationship to reward and self-administration of playback. J. Comp. Psychol. 122, 357–367. 10.1037/a0012889 19014259

[B6] Byers S. L. Wiles M. V. Dunn S. L. Taft R. A. (2012). Mouse estrous cycle identification tool and images. PLoS One 7:e35538. 10.1371/journal.pone.0035538 22514749 PMC3325956

[B7] Carola V. D’Olimpio F. Brunamonti E. Mangia F. Renzi P. (2002). Evaluation of the elevated plus-maze and open-field tests for the assessment of anxiety-related behaviour in inbred mice. Behav. Brain Res. 134, 49–57. 10.1016/s0166-4328(01)00452-1 12191791

[B8] Chabout J. Sarkar A. Dunson D. B. Jarvis E. D. (2015). Male mice song syntax depends on social contexts and influences female preferences. Front. Behav. Neurosci. 9:76. 10.3389/fnbeh.2015.00076 25883559 PMC4383150

[B9] Chen Q. Panksepp J. B. Lahvis G. P. (2009). Empathy is moderated by genetic background in mice. PLoS One 4:e4387. 10.1371/journal.pone.0004387 19209221 PMC2633046

[B10] Critchlow V. Liebelt R. A. Bar-Sela M. Mountcastle W. Lipscomb H. S. (1963). Sex difference in resting pituitary-adrenal function in the rat. Am. J. Physiol. 205, 807–815. 10.1152/ajplegacy.1963.205.5.807 4291060

[B11] Demir E. Li K. Bobrowski-Khoury N. Sanders J. I. Beynon R. J. Hurst J. L.. (2020). The pheromone darcin drives a circuit for innate and reinforced behaviours. Nature 578, 137–141. 10.1038/s41586-020-1967-8 31996852

[B12] Gadziola M. A. Grimsley J. M. S. Faure P. A. Wenstrup J. J. (2012). Social vocalizations of big brown bats vary with behavioral context. PLoS One 7:e44550. 10.1371/journal.pone.0044550 22970247 PMC3436781

[B13] Gadziola M. A. Shanbhag S. J. Wenstrup J. J. (2016). Two distinct representations of social vocalizations in the basolateral amygdala. J. Neurophysiol. 115, 868–886. 10.1152/jn.00953.2015 26538612 PMC4839489

[B14] Gaub S. Fisher S. E. Ehret G. (2016). Ultrasonic vocalizations of adult male Foxp2 -mutant mice: behavioral contexts of arousal and emotion. Genes Brain Behav. 15, 243–259. 10.1111/gbb.12274 26566793

[B15] Ghasemahmad Z. Panditi R. Bhavya S. Drishna K. Wenstrup J. J. (2020). Emotional vocalizations in mice evoke distinct patterns of dopamine and acetylcholine release in the amygdala after brief mating and restraint experiences. Assoc. Res. Ontol. Abstr. 43:480.

[B16] Ghasemahmad Z. Sharma B. Perumal D. K. Panditi R. Wenstrup J. J. (2019). Valence of mating but not restraint vocalizations is perceived differently by male and female mice. Assoc. Res. Ontol. Abstr. 42:498.

[B17] Gourbal B. F. Barthelemy M. Petit G. Gabrion C. (2004). Spectrographic analysis of the ultrasonic vocalisations of adult male and female BALB/c mice. Naturwissenschaften 91, 381–385. 10.1007/s00114-004-0543-7 15278217

[B18] Grimsley J. M. S. Hazlett E. G. Wenstrup J. J. (2013). Coding the meaning of sounds: contextual modulation of auditory responses in the basolateral amygdala. J. Neurosci. 33, 17538–17548. 10.1523/JNEUROSCI.2205-13.2013 24174686 PMC3812514

[B19] Grimsley J. M. S. Monaghan J. J. M. Wenstrup J. J. (2011). Development of social vocalizations in mice. PLoS One 6:e17460. 10.1371/journal.pone.0017460 21408007 PMC3052362

[B20] Grimsley J. M. S. Sheth S. Vallabh N. Grimsley C. A. Bhattal J. Latsko M.. (2016). Contextual modulation of vocal behavior in mouse: newly identified 12 khz “mid-frequency” vocalization emitted during restraint. Front. Behav. Neurosci. 10:38. 10.3389/fnbeh.2016.00038 27014000 PMC4783392

[B21] Hammerschmidt K. Radyushkin K. Ehrenreich H. Fischer J. (2009). Female mice respond to male ultrasonic “songs” with approach behaviour. Biol. Lett. 5, 589–592. 10.1098/rsbl.2009.0317 19515648 PMC2781958

[B22] Handa R. J. Burgess L. H. Kerr J. E. O’Keefe J. A. (1994). Gonadal steroid hormone receptors and sex differences in the hypothalamo-pituitary-adrenal axis. Horm. Behav. 28, 464–476. 10.1006/hbeh.1994.1044 7729815

[B23] Hanson J. L. Hurley L. M. (2012). Female presence and estrous state influence mouse ultrasonic courtship vocalizations. PLoS One 7:e40782. 10.1371/journal.pone.0040782 22815817 PMC3399843

[B24] Heckman J. J. Proville R. Heckman G. J. Azarfar A. Celikel T. Englitz B. (2017). High-precision spatial localization of mouse vocalizations during social interaction. Sci. Rep. 7:3017. 10.1038/s41598-017-02954-z 28592832 PMC5462771

[B25] Holy T. E. Guo Z. (2005). Ultrasonic songs of male mice. PLoS Biol. 3:e386. 10.1371/journal.pbio.0030386 16248680 PMC1275525

[B26] Jasnow A. M. Huhman K. L. Bartness T. J. Demas G. E. (2000). Short-day increases in aggression are inversely related to circulating testosterone concentrations in male siberian hamsters (*Phodopus sungorus*). Horm. Behav. 38, 102–110. 10.1006/hbeh.2000.1604 10964524

[B27] Keesom S. M. Hurley L. M. (2016). Socially induced serotonergic fluctuations in the male auditory midbrain correlate with female behavior during courtship. J. Neurophysiol. 115, 1786–1796. 10.1152/jn.00742.2015 26792882 PMC4869479

[B28] Kitay J. I. (1961). Sex differences in adrenal cortical secretion in the rat. Endocrinology 68, 818–824. 10.1210/endo-68-5-818 13756461

[B29] Knutson B. Burgdorf J. Panksepp J. (2002). Ultrasonic vocalizations as indices of affective states in rats. Psychol. Bull. 128, 961–977. 10.1037/0033-2909.128.6.961 12405139

[B30] Kulesskaya N. Voikar V. (2014). Assessment of mouse anxiety-like behavior in the light-dark box and open-field arena: role of equipment and procedure. Physiol. Behav. 133, 30–38. 10.1016/j.physbeh.2014.05.006 24832050

[B31] Lahvis G. P. Alleva E. Scattoni M. L. (2011). Translating mouse vocalizations: prosody and frequency modulation1. Genes Brain Behav. 10, 4–16. 10.1111/j.1601-183X.2010.00603.x 20497235 PMC2936813

[B32] Liu R. C. Miller K. D. Merzenich M. M. Schreiner C. E. (2003). Acoustic variability and distinguishability among mouse ultrasound vocalizations. J. Acoust. Soc. Am. 114, 3412–3422. 10.1121/1.1623787 14714820

[B33] Maggio J. C. Whitney G. (1985). Ultrasonic vocalizing by adult female mice (*mus musculus*). J. Comp. Psychol. 99, 420–436. 10.1037/0735-7036.99.4.420 4075780

[B34] Maney D. L. (2013). The incentive salience of courtship vocalizations: hormone-mediated “wanting” in the auditory system. Hear. Res. 305, 19–30. 10.1016/j.heares.2013.04.011 23665125

[B35] Moles A. Costantini F. Garbugino L. Zanettini C. D’Amato F. R. (2007). Ultrasonic vocalizations emitted during dyadic interactions in female mice: a possible index of sociability? Behav. Brain Res. 182, 223–230. 10.1016/j.bbr.2007.01.020 17336405

[B36] Musolf K. Hoffmann F. Penn D. J. (2010). Ultrasonic courtship vocalizations in wild house mice, mus musculus musculus. Anim. Behav. 79, 757–764. 10.1016/j.anbehav.2009.12.034

[B37] Neunuebel J. P. Taylor A. L. Arthur B. J. Egnor S. E. R. (2015). Female mice ultrasonically interact with males during courtship displays. eLife 4:e06203. 10.7554/eLife.06203 26020291 PMC4447045

[B38] Nyby J. (1983). Ultrasonic vocalizations during sex behavior of male house mice (mus musculus): a description. Behav. Neural Biol. 39, 128–134. 10.1016/s0163-1047(83)90722-7 6661142

[B39] Ohlemiller K. K. Dahl A. R. Gagnon P. M. (2010). Divergent aging characteristics in CBA/J and CBA/CaJ mouse cochleae. J. Assoc. Res. Otolaryngol. 11, 605–623. 10.1007/s10162-010-0228-1 20706857 PMC2975886

[B40] Oyola M. G. Handa R. J. (2017). Hypothalamic-pituitary-adrenal and hypothalamic-pituitary-gonadal axes: sex differences in regulation of stress responsivity. Stress 20, 476–494. 10.1080/10253890.2017.1369523 28859530 PMC5815295

[B41] Pomerantz S. M. Nunez A. A. Bean N. J. (1983). Female behavior is affected by male ultrasonic vocalizations in house mice. Physiol. Behav. 31, 91–96. 10.1016/0031-9384(83)90101-4 6685321

[B42] Portfors C. V. (2007). Types and functions of ultrasonic vocalizations in laboratory rats and mice. J. Am. Assoc. Lab. Anim. Sci. 46, 28–34. 17203913

[B43] Rotschafer S. E. Trujillo M. S. Dansie L. E. Ethell I. M. Razak K. A. (2012). Minocycline treatment reverses ultrasonic vocalization production deficit in a mouse model of fragile x syndrome. Brain Res. 1439, 7–14. 10.1016/j.brainres.2011.12.041 22265702

[B44] Sadananda M. Wöhr M. Schwarting R. K. W. (2008). Playback of 22-kHz and 50-kHz ultrasonic vocalizations induces differential c-fos expression in rat brain. Neurosci. Lett. 435, 17–23. 10.1016/j.neulet.2008.02.002 18328625

[B45] Sales née Sewell G. D. (1972). Ultrasound and mating behaviour in rodents with some observations on other behavioural situations. J. Zool. 168, 149–164. 10.1111/j.1469-7998.1972.tb01345.x

[B46] Sangiamo D. T. Warren M. R. Neunuebel J. P. (2020). Ultrasonic signals associated with different types of social behavior of mice. Nat. Neurosci. 23, 411–422. 10.1038/s41593-020-0584-z 32066980 PMC7065962

[B47] Scattoni M. L. Crawley J. Ricceri L. (2009). Ultrasonic vocalizations: a tool for behavioural phenotyping of mouse models of neurodevelopmental disorders. Neurosci. Biobehav. Rev. 33, 508–515. 10.1016/j.neubiorev.2008.08.003 18771687 PMC2688771

[B48] Scattoni M. L. Gandhy S. U. Ricceri L. Crawley J. N. (2008). Unusual repertoire of vocalizations in the BTBR T+tf/J mouse model of autism. PLoS One 3:e3067. 10.1371/journal.pone.0003067 18728777 PMC2516927

[B49] Seibenhener M. L. Wooten M. C. (2015). Use of the Open Field Maze to measure locomotor and anxiety-like behavior in mice. J. Vis. Exp. 96:e52434. 10.3791/52434 25742564 PMC4354627

[B50] Sisneros J. A. Forlano P. M. Deitcher D. L. Bass A. H. (2004). Steroid-dependent auditory plasticity leads to adaptive coupling of sender and receiver. Science 305, 404–407. 10.1126/science.1097218 15256672

[B51] Smith J. (1965). The evolution of alarm calls. Am. Nat. 99, 59–63. 10.1086/282349

[B52] Tsukano H. Horie M. Bo T. Uchimura A. Hishida R. Kudoh M.. (2015). Delineation of a frequency-organized region isolated from the mouse primary auditory cortex. J. Neurophysiol. 113, 2900–2920. 10.1152/jn.00932.2014 25695649 PMC4416634

[B53] Wang H. Liang S. Burgdorf J. Wess J. Yeomans J. (2008). Ultrasonic vocalizations induced by sex and amphetamine in M2, M4, M5 muscarinic and D2 dopamine receptor knockout mice. PLoS One 3:e1893. 10.1371/journal.pone.0001893 18382674 PMC2268741

[B54] Wenstrup J. J. Ghasemahmad Z. Hazlett E. Shanbhag S. J. (2020). “The amygdala—a hub of the social auditory brain,” in The Senses: A Comprehensive Reference, ed. Fritzsch B. (San Diego, CA: Elsevier Science Publishing Co. Inc.).

[B55] Williams W. O. Riskin D. K. Mott A. K. M. (2008). Ultrasonic sound as an indicator of acute pain in laboratory mice. J. Am. Assoc. Lab. Anim. Sci. 47, 8–10. 18210991 PMC2652617

[B57] Wöhr M. Roullet F. I. Crawley J. N. (2011). Reduced scent marking and ultrasonic vocalizations in the BTBR T+tf/J mouse model of autism. Genes Brain Behav. 10, 35–43. 10.1111/j.1601-183X.2010.00582.x 20345893 PMC2903641

[B56] Wöhr M. Schwarting R. K. W. (2013). Affective communication in rodents: ultrasonic vocalizations as a tool for research on emotion and motivation. Cell Tissue Res. 354, 81–97. 10.1007/s00441-013-1607-9 23576070

